# Current Status of Clinical and Genetic Screening of Hereditary Hemorrhagic Telangiectasia Families in Hungary

**DOI:** 10.3390/jcm10173774

**Published:** 2021-08-24

**Authors:** Tamás Major, Zsuzsanna Bereczky, Réka Gindele, Gábor Balogh, Benedek Rácz, László Bora, Zsolt Kézsmárki, Boglárka Brúgós, György Pfliegler

**Affiliations:** 1 Division of Otorhinolaryngology and Head & Neck Surgery, Kenézy Gyula Campus, University of Debrecen Medical Center, University of Debrecen, H-4031 Debrecen, Hungary; benedekrcz@gmail.com; 2 Division of Clinical Laboratory Science, Department of Laboratory Medicine, Faculty of Medicine, University of Debrecen, H-4032 Debrecen, Hungary; gindele.reka@med.unideb.hu (R.G.); balogh.gabor@med.unideb.hu (G.B.); 3 Department of Radiology, Szent Lázár County Hospital, H-3100 Salgótarján, Hungary; crisicum@gmail.com; 4 Division of Radiology, Kenézy Gyula Campus, University of Debrecen Medical Center, University of Debrecen, H-4031 Debrecen, Hungary; kezsmarki.zsolt@kenezy.unideb.hu; 5 Division of Rare Diseases, Department of Internal Medicine Block B, Faculty of Medicine, University of Debrecen, H-4032 Debrecen, Hungary; brugosb@med.unideb.hu (B.B.); pfliegler@med.unideb.hu (G.P.)

**Keywords:** hereditary hemorrhagic telangiectasia, ENG, ACVRL1, SMAD4, germline mutation, genetic test, Curaçao criteria, family screening

## 1. Introduction

Hereditary hemorrhagic telangiectasia (HHT) is a rare germline vascular malformation syndrome with a prevalence of 1:5000–1:10,000 [1,2]. HHT is listed in both the capillary (telangiectasia subgroup) and the arteriovenous malformation (AVM) groups in the 2018 classification from the International Society for the Study of Vascular Anomalies (ISSVA) [3]. Due to its complexity, HHT appears relatively late as a syndrome in medical history, starting with the fundamental papers of Rendu, Osler and Weber, published between 1896 and 1907 [2].

Telangiectases are mucocutaneous (1–2 mm in diameter), and AVMs are high-flow, solid organ arteriovenous shunts bypassing the intervening capillary bed [4]. Both telangiectases and AVMs show very characteristic localizations in HHT, reflected by the Curaçao criteria: 1. spontaneous, recurrent nosebleeds; 2. multiple telangiectases at characteristic sites (lips, oral cavity, fingers and nose); 3. visceral lesions such as gastrointestinal telangiectasia with or without bleeding including pulmonary, hepatic and cerebral AVMs (PAVMs, HAVMs and CAVMs, respectively); and 4. a first degree relative with HHT according to these criteria [5]. Major acute or chronic complications might be deduced through shunting (dyspnea, ischaemic strokes and brain abscesses by pulmonary right-to-left shunting, pulmonary hypertension and high output cardiac failure by left-to-right hepatic shunting, portal hypertension by hepatoportal shunting, encephalopathy by portohepatic shunting) and bleeding (hemorrhagic strokes, hemoptysis and anemia due to epistaxis or gastrointestinal bleeding) [6].

The majority of familial (germline) vascular malformations or syndromes including HHT are inherited in an autosomal-dominant trait with age-dependent penetrance, and mutations are usually family-specific [1]. Causative genes identified to date are ENG (encodes endoglin; mutations account for the HHT1 phenotype) [7] and ACVRL1 (encodes activin receptor-like kinase 1; mutations account for the HHT2 phenotype) [8] in 85% of HHT families, SMAD4, accounting for the juvenile polyposis–HHT phenotype, in 2% of HHT families [9] and GDF2 (encodes bone morphogenic protein 9; mutations account for the extremely rare HHT5 phenotype) [10]. The phenotypic spectrum of HHT1 and HHT2 is slightly different, with an earlier onset of symptoms in HHT1, more PAVMs and CAVMs in HHT1 and more HAVMs in HHT2 [6], resulting in a generally more severe phenotype in HHT1. Each protein encoded by the above genes belongs to the transforming growth factor-beta (TGF-β) superfamily controlling angiogenesis. All variant types (missense, nonsense, splice-site, frameshift, in-frame deletions and insertions and finally, large deletions and insertions in 10% of cases) have been described throughout the ENG, ACVRL1 and SMAD4 [11]. On the other hand, the HHT Mutation Database on the Associated Regional and University Pathologists (ARUP) laboratory’s website [12] enumerates 510 ENG and 572 ACVRL1 variants at present.

Being aware of the most frequently affected genes and their allele heterogeneity, the proposed molecular testing algorithm for HHT is ENG and ACVRL1 sequencing and a large deletion–insertion test (through multiplex ligation-dependent probe amplification, MLPA) followed by SMAD4 sequencing as well as MLPA if no variants of certain pathogenicity are found in the ENG and ACVRL1 genes [11,13].

The diagnosis of HHT remains a challenge, especially in probands. In a comprehensive study of 233 patients recruited between 2000 and 2009 [14], the diagnostic time lag (the interval between the first symptoms and the diagnosis of HHT) was 29.1 years for index cases and 22.6 years for non-index cases. Its first period was the interval between disease onset and the patient’s referral to any physicians due to HHT-related manifestations (called the referral time lag), explained by the authors as being due to the poor knowledge of HHT in society. The long second period between the patients’ referrals to a diagnosis of HHT is attributable to the unawareness of HHT within the medical community [14]. HHT is a rare disease with age-related penetrance of its multisystemic symptoms, and in addition, congenital PAVMs and CAVMs are most often asymptomatic until emerging as severe, acute complications in a subset of patients. The involved disciplines might address the symptoms one by one, often without the chance to assemble the underlying syndrome. At the University of Debrecen and the surrounding hospitals in Northeast Hungary, the management of HHT patients started a decade ago. The objective of this study is to give an account of the current status of the Hungarian HHT families’ clinical and genetic screenings, performed in order to reduce the diagnostic time lag of the disease.

## 2. Patients and Methods

### 2.1. Patient Recruitment

The initial physical examinations were performed at the Division of Rare Diseases, Faculty of Medicine, University of Debrecen, in the Division of Otorhinolaryngology and Head and Neck Surgery, Kenézy Gyula Campus, University of Debrecen Medical Center; in the Department of Otorhinolaryngology, Ferenc Markhot County Hospital, Eger; and in the Department of Otolaryngology and Head and Neck Surgery, Borsod-Abaúj-Zemplén County Central Hospital and University Teaching Hospital, Miskolc, Hungary by the internist (G.P. and B.B.) and otorhinolaryngologist (T.M.) authors, respectively, all experts in HHT within their specialties. Patients with known or suspected HHT with habitation throughout Hungary presented themselves or were referred by their family doctors or specialists. The only well-defined denominator population involved in the study was the primary attendance area of the Ferenc Markhot County Hospital, Eger, Hungary (population of 225,339), where the stratified population screening of HHT was executed [15].

### 2.2. Clinical Evaluation

Our clinical HHT examination protocol started with a thorough medical history (nosebleeds, telangiectases, dyspnea, stroke, migraine, brain abscess, abdominal pain, anemia, hemoptysis, melena, etc. as well as family history concerning the same) as well as ENT and internal (dyspnea, clubbing, hepatic bruits, etc.) physical examinations completed through the evaluation of each characteristic telangiectasis site.

Adult probands fulfilling at least 2 Curaçao criteria (mostly epistaxis, telangiectases or a first degree relative with HHT) underwent a visceral AVM screening through simultaneous non-enhanced and contrast-enhanced, arterial and venous phase chest and upper abdominal computed tomography (CT) (Siemens Somatom Definition AS 64; Siemens Shanghai Medical Equipment, Shanghai, China) ) as well as a magnetic resonance (MR) examination (Siemens Magnetom Essenza 1.5 T; Siemens Shenzhen Magnetic Resonance, Shenzhen, China) of the brain following the “vascular malformation” protocol with T1 sagittal, T2 axial and T2 fluid-attenuated inversion recovery (FLAIR) coronal, diffusion-weighted imaging (DWI); susceptibility-weighted imaging (SWI); non-enhanced and contrast-enhanced 3D time-of-flight (TOF) angiography and postcontrast 3DT1 examinations. If patients underwent contrast-enhanced chest CT or brain MR with any other indications within three years prior to enrollment to our study, images were reassessed by the radiologists in our study group (L.B. and Z.K.) in order to reduce evaluation bias. Endoscopic examination of the upper or lower digestive tracts was offered at the suspicion of gastrointestinal bleeding or in the case of long-standing anemia disproportionate to epistaxis. Laboratory tests including complete blood count, iron status and liver function were recommended at the first visit. Pedigree charts were constructed using information from probands and senior family members. Proband evaluation was accomplished using genetic testing.

In pediatric patients under 18 years of age, a chest radiograph as well as pulse oximetry in supine (considered abnormal if SaO2 < 96%) and erect positions (abnormal if it decreased by ≥2%) was performed to screen for PAVMs. MR examination for CAVMs was offered to symptomatic children; otherwise, it was postponed until adulthood.

For at-risk family members, physical examinations were also performed. In the case of definite or suspected HHT, evaluations proceeded with the visceral AVM screening protocol. Genetic screening for the causative, family-specific mutation was offered for each at-risk family member, regardless of HHT status. If a family member’s HHT status was clinically evaluated as “unlikely” but the family-specific mutation was detected (especially in younger individuals), the patient underwent the AVM screening.

### 2.3. Mutation Analysis

The isolation of genomic DNA from peripheral, citrated whole blood, and Sanger sequencing of the exons and flanking intronic sites of ENG, ACVRL1 and SMAD4 were performed as previously reported [16,17]. Three probands were tested using next-generation sequencing covering HHT causative genes (ACVRL1, ENG, SMAD4 and GDF2), among others [17].

In cases where no mutation was found through Sanger sequencing, MLPA analysis was performed using a SALSA MLPA Kit P093 HHT/HPAH (MRC-Holland, Amsterdam, The Netherlands). The MLPA data were analyzed using Coffalyser.Net Software (version 140721.1958, MRC-Holland, Amsterdam, The Netherlands, 2021).

### 2.4. Variant Assessment

Following screening for polymorphisms in the dbSNP and 1000 Genomes databases and in randomly selected, healthy control individuals (*n* = 50) from the framework of the Hungarian General Practitioners’ Morbidity Sentinel Stations Program (HMSSP), representing the general Hungarian population [18], variants were verified in the Human Gene Mutation Database (HGMD) [19] and the Associated Regional and University Pathologist (ARUP) Mutation Database [12]. Novel variants were tested for familial cosegregation when several affected and non-affected at-risk family members were available. To assess the probability of the cosegregation of the variant with disease, the simplified method for cosegregation analysis (SISA) [20] was used. The pathogenicity of novel missense variants was assessed using Polyphen2 HumDiv and HumVar, MutPred2 and SIFT in silico prediction-modeling software and with the Franklin Genoox platform [21]. In the case of novel splice-site variants, the Human Splicing Finder software was used. Finally, variants were classified as pathogenic, likely pathogenic or variants of uncertain significance (VUS) on the basis of databases (known variants) or the standards and guidelines of the American College of Medical Genetics and Genomics (ACMG) [22].

### 2.5. The Algorithm of Cascade Family Screening

**Figure 1:**
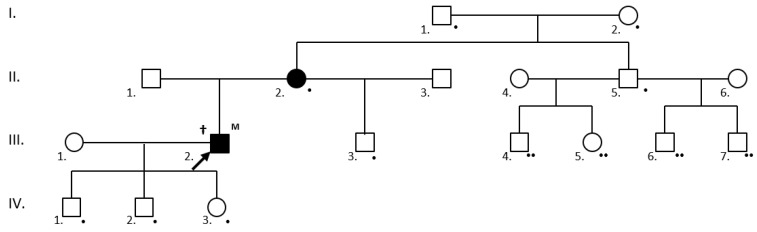
Cascade mutation screening in a family with a definite HHT (†) III.2. proband (arrow) with a novel, likely pathogenic ENG mutation (M). The following individuals were obligately testable (•): the IV.1., IV.2. and IV.3. first-degree asymptomatic relatives of the mutation carrier; the II.2. clinically affected first-degree relatives of the mutation carrier; and the III.3. (son), II.5. (sibling), I.1. and I.2. (parents) first-degree relatives of the affected II.2. individual. Individuals III.4., III.5., III.6 and III.7. became testable (facultatively testable at present) (••) if their father proved to be a mutation carrier at the obligate testing. Clinically and/or genetically affected individuals are shaded.

At-risk individuals awaiting screening in families with known mutations were classified as obligately or facultatively testable. The obligately testable family members were 1. first degree relatives of individuals positive for the family-specific mutation and 2. patients fulfilling ≥2 Curaçao criteria (suspected or definite HHT) and their first-degree relatives. The facultatively testable individuals were all asymptomatic first-degree relatives of individuals who tested positive in the obligate group (Figure 1).

### 2.6. Statistical Analysis

The Kolmogorov–Smirnov test and Shapiro–Wilk test were performed to examine the normality of age distribution. Results regarding the continuous variable of age were expressed as mean ± SD. Between-group differences in age were analyzed using the Student’s *t*-test. Differences in category frequencies were evaluated using the χ2 test. A *p*-value of 0.05 or less was considered to indicate statistical significance. All statistical analyses were performed using the Statistical Package for the Social Sciences (SPSS 26.0), Chicago, IL, USA, 2021.

## 3. Results

### 3.1. Demographical Data

**Figure 2:**
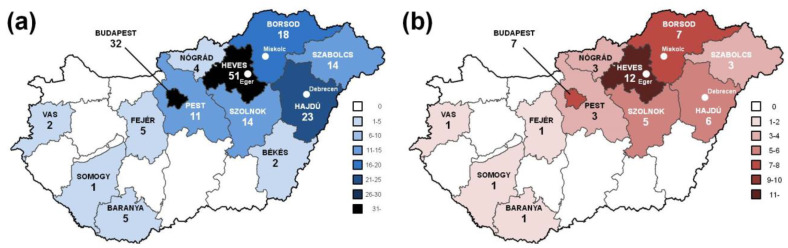
Distribution of all individuals (**a**) and probands (**b**) tested for HHT according to habitation in the 19 Hungarian counties as well as Budapest. The clinical and genetic HHT-testing institutions are indicated with white dots.

Including probands and at-risk family members, a total of 186 individuals of Hungarian ethnicity (84 males and 102 females) in 50 families (18 male and 32 female probands aged 56.5 ± 12.9 years; 66 male and 70 at-risk family members aged 35.8 ± 19.2 years) were evaluated for HHT using our clinical and genetic algorithm. One hundred and eighty-two members of the study cohort had a habitation in Hungary, with a predominance in the northeast at the time of the test (Figure 2a); the remainder were living in Austria, Germany, Great Britain and Italy. Probands’ habitation (Figure 2b) followed a similar geographical distribution. In 24% of kindreds (12/50) only the probands were tested, while in 42% (21/50) 2–3 individuals, in 18% (9/50) 4–6 individuals, in 10% (5/50) 7–10 individuals and finally, in 6% (3/50) > 10 individuals were tested, giving a rate of 3.72 individuals/family.

### 3.2. Mutation Analysis

**Figure 3:**
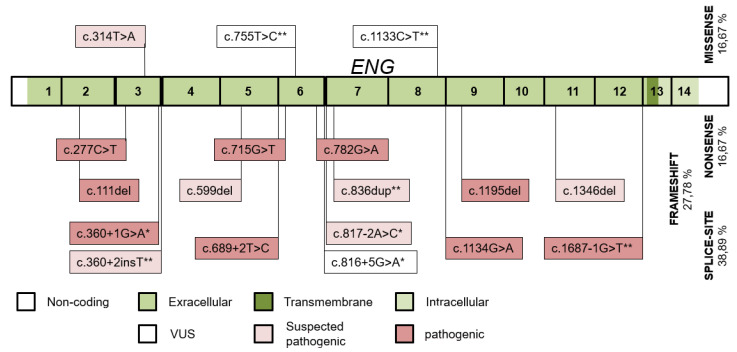
The distribution of ENG variants detected throughout the ENG exons and their intronic boundaries. Variants published by our study group and novel variants in this study are indicated with (*) and (**), respectively.

A mutation was identified in 48 of the 50 families, giving a mutation detection rate of 96%. Eighteen different ENG mutations were detected in 53 individuals of 21 families (Figure 3). Fifteen variants were pathogenic or likely pathogenic, and 3 were VUS (the c.816+5G>A variant has a ≥99.22% probability of cosegregation by SISA). Thirteen of the ENG variants were published (3 of them by our study group) [23]. Splice-site and frameshift variants occurred most frequently. Two variants were each detected in two apparently unrelated families. The wild-type ENG allele was detected in 31 individuals.

**Figure 4:**
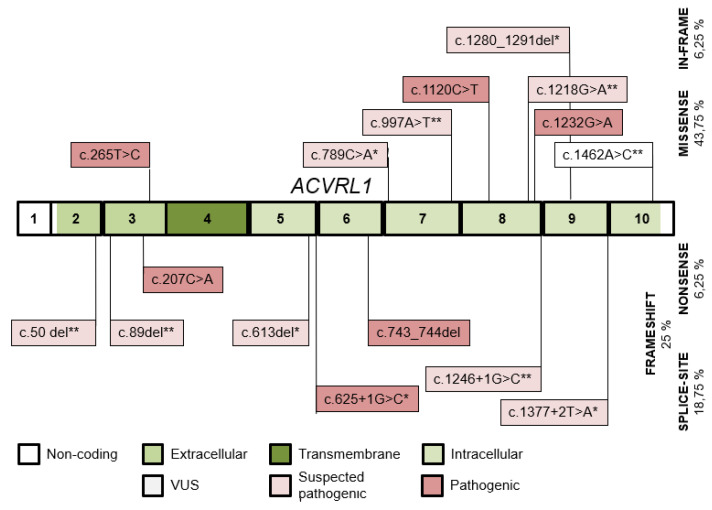
The distribution of ACVRL1 variants detected throughout the ACVRL1 exons and their intronic boundaries. Variants published by our study group and novel variants in this study are indicated with (*) and (**), respectively.

Sixteen ACVRL1 variants (Figure 4) were detected in 63 individuals of 26 kindreds, 10 of them were published (5 of them by our study group) [23]. Variants were pathogenic or likely pathogenic with the exception of 2 VUS. Three variants were shared by 2, 3 and 8 families. The predominant mutation type was missense. The wild-type ACVRL1 allele was detected in 34 individuals.

Thus, the ENG/ACVRL1 mutation rate was 1.13 (18/16) and the ENG/ACVRL1 family rate was 0.81 (21/26), while the ENG/ACVRL1 mutation positive individuals’ rate was 0.84 (53/63).

A SMAD4 c.7A>G variant was identified in an additional kindred [17]. In the remaining two families (with 4 affected individuals) no mutation was detected despite performing the ENG, ACVRL1 and SMAD4 exon and flanking intronic sequencing and MLPA tests in the probands with definite HHT.

The hitherto unpublished variants and their evaluations of pathogenicity are detailed in Table 1.

### 3.3. Genotype–Phenotype Correlations

Patient numbers and gender rates in the overall (*n* = 116) HHT1 + HHT2 cohorts and within their subdivisions based upon their fulfilled Curaçao criteria are shown in Table 2. Interestingly, in the definite HHT subgroup, the number of females was significantly higher among HHT2 compared to HHT1 patients (*p* = 0.020). This difference remained significant concerning the overall HHT2 vs HHT1 cohorts (*p* = 0.040). Five asymptomatic individuals (0–33 years of age) carried the family-specific ACVRL1 mutation. In addition, in 31 (12 males and 19 females, aged 35.6 ± 18.3 years) and 34 (17 males and 17 females, aged 35.1 ± 21.5 years) unaffected individuals of the HHT1 and HHT2 kindreds, respectively, the wild-type ENG and ACVRL1 alleles were detected.

All probands and at-risk family members underwent the ENT and internal physical examination protocols. However, the visceral AVM screening was incomplete at the time of writing the manuscript in a subset of patients, while others refused the imaging studies. The results of the clinical evaluations are shown in Table 3.

Epistaxis was observed in all-but-one definite HHT1 + HHT2 patients. The exception was a 34-year-old HHT2 female with mucocutaneous telangiectases at characteristic sites, HAVM and a positive family history. Nosebleeds were significantly more common in the overall HHT1 cohort. Telangiectasia was more common in HHT2 patients in the overall HHT cohort, and this difference was significant in the definite subgroup. In the suspected HHT subgroup, all patients had epistaxis (90%) or telangiectasia (10%) as the second criterion added to their family histories.

**Figure 5:**
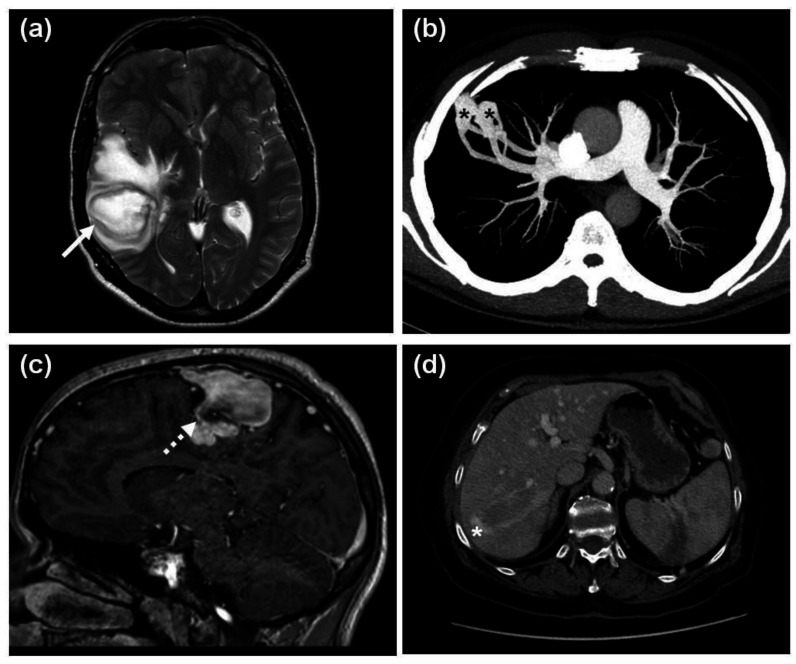
Visceral manifestations of HHT. (**a**) An axial plane, T2-weighted MR image of a cerebral abscess (white arrow) in the right temporal lobe of a 43-year-old HHT1 male and (**b**) the axial plane maximum intensity projection (MIP) CT image of the underlying 20 mm and 16 mm PAVMs (black asterisks) and their feeding vessels in the periphery of the right S3 segment in the same patient; (**c**) A 3DT1 postcontrast sagittal plane image of a CAVM (white dotted arrow) in the right parietal lobe of a 21-year-old male with HHT1; (**d**) An axial plane venous phase CT image showing a curved portovenous HAVM (white asterisk) with feeding vessels in the right liver lobe of a 73-year-old female HHT2 patient.

Concerning visceral lesions, PAVMs were significantly more common in the HHT1 group. Four patients (3 with HHT1 and 1 with HHT2) had cerebral abscess. A HHT2 patient died of this at the age of 42 years. He had recurrent nosebleeds and mucocutaneous telangiectases, but he was not tested for HHT when alive. One year prior to his death, he was diagnosed with polycythemia (hemoglobin 20.6 g/dL) and tested for the JAK2 V617F variant with a negative result. A chest radiogram did not show any soft-tissue opacities, but CT was not performed. No pulmonary masses were described in his autopsy report. As the family-specific ACVRL1 c.50del was detected in his mother (proband with definite HHT) and daughter (with suspected HHT) six years later, his preserved DNA was also screened for this variant, and the mutation was detected. The three HHT1 patients with cerebral abscess had a detectable causative PAVM (Figure 5a,b) and in two of them, the cerebral abscess preceded the diagnosis of PAVM. The third HHT1 patient was a 22-year-old male with clubbing, dyspnea on exertion, polycythemia and a large pulmonary soft-tissue mass at onset, resulting in a long-term differential diagnostic pitfall [24]. In summary, the prevalence of cerebral abscess in the cohort with unambiguous PAVM and the overall HHT1 + HHT2 cohorts was 12.5% (3/24) and 3.4% (4/116), respectively.

CAVMs were shown in 8.8% of all HHT patients undergoing brain MR with a near significant (*p* = 0.056) predominance in HHT1. Three of them were asymptomatic, and one had a chronic headache (Figure 5c). HAVMs (Figure 5d) were significantly more common in HHT2. Neither physical signs (bruits, palpable masses) nor comorbidities (portal hypertension, high output cardiac failure, hepatic encephalopathy, etc.) unambiguously associated with HAVMs were observed. Symptomatic gastrointestinal telangiectases were detected in 13.8% of the overall HHT cohort, with a mild HHT1 predominance.

Within our HHT1 + HHT2 cohort, 24 individuals were minors. Their results are detailed in Table 4. Among the 9 patients with pathogenic or likely pathogenic variants, the leading symptom was epistaxis in 5 cases, followed by telangiectases in 2 cases. Considering visceral lesions, only PAVM screening was performed with negative results; otherwise, no patients were found with history or symptoms suggesting cerebral or hepatic AVMs. Fifteen individuals showed the wild-type ENG and ACVRL1 alleles.

A novel SMAD4 c.7A>G variant classified as a VUS was found in a 64-year-old female. She had recurrent nosebleeds, oral and lower lip telangiectases and two first-degree relatives with epistaxis. Neither the patient nor the kindred had a history of JPS, gastric or colon cancers. She had no anemia. Endoscopic examinations of the upper and lower gastrointestinal tract were offered, but she refused these. Her family was unavailable for clinical and genetic screening as well. On the basis of her incomplete evaluation, we could not determine whether she had a pure HHT or a JP–HHT phenotype.

### 3.4. Cascade Family Screening

Pedigree charts were constructed in 32 kindreds with 23 ENG/ACVRL1 variants (20 of them pathogenic or likely pathogenic in addition to 3 VUS). Table 5 shows the momentary states of the cascade mutation screening. The HHT mutation status was clarified in 156 individuals, 24 refused testing, an additional 151 were obligately testable, and 26 were facultatively testable.

## 4. Discussion

At present, we have 186 clinically and genetically tested individuals from 50 families (with provenience to the northeast in the majority of cases) at our HHT Centre in Debrecen, Hungary, corresponding to an individual/family rate of 3.72 and a patient/family rate of 2.42. These data are comparable with other studies evaluating a minimum of 100 HHT patients, ranging from 3.74 to 4.52 and 2.08 to 4.93, respectively (Table 6). In accordance with an autosomal dominant disease, the male-to-female rate in the overall HHT cohort was approximately equal (55/63). The only exception was the definite HHT2 cohort, in which a pronounced female predominance was observed (male-to-female rate of 19/33). Several studies report a female preponderance among patients diagnosed with HHT [25,26] as well as among patients identified through the analysis of HHT medical codes from a computerized general practice database yielding a representative sample of the UK population [27] or through combinations strongly suggestive of HHT in a US health insurance database [28]. This gender bias has been explained through behavioral factors (wider access of women to healthcare resources needed due to contraception, pregnancy and childcare) and biological factors [27].

All of the above HHT studies and the vast majority of papers in the literature are from Europe or North America, resulting in a publication bias. The methods of ENG, ACVRL1 and SMAD4 variant detection differ, and in particular, earlier studies lacked large deletion/insertion [4,36] or SMAD4 testing [4,29,36], accounting for approximately 10% and 2% of all HHT mutations, respectively [11]. In addition, the mutation detection rate is affected by the proband selection for the test, too. The strict application of the Curaçao criteria in probands (i.e., fulfillment of at least 3 criteria) resulted in an ENG and ACVRL1 mutation detection rate of 96.1% in a recent study [38], matching ours. Among our probands, only four were evaluated as having suspected HHT, but their AVM screenings were incomplete. All of them had a likely pathogenic ENG or ACVRL1 variant. The other 48 probands (including the 2 without detectable mutations) were definite HHT patients.

The “ENG/ACVRL1 rate” affecting HHT phenotype in a population is often described with some inaccuracy in the literature. In our opinion, this rate consists of three components: First, the ENG/ACVRL1 mutation rate shows considerable variation in different populations ranging from 0.52 to 1.69, even in larger cohorts (Table 6). This rate reflects the real allele heterogeneity of a given population, distorted by a potential referral bias associated with the HHT phenotype [35]. Second, in addition to this referral bias, the ENG/ACVRL1 proband (family) rate (ranging from 0.40 to 1.58) is further distorted by founder effects [39]. Three ACVRL1 founder variants detected in 24, 7 and 5 families accounted for 37.1% (36/97) of all mutation-positive kindreds in the Norwegian national HHT study [30]. The French founder variant (ACVRL1 c.1112_1113dupG) was detected in 17% (17/100) of families in the French HHT network [31]; four years later, 35 families were known to have this variant [40]. The Danish founder (ENG c.360C>A) accounted for 13.7% (13/95) of all family-specific variants in a national study [13]. In each of the above variants, the founder effect was confirmed by haplotype analysis. Third, the ENG/ACVRL1 patient rate might be further biased by the efficacy of family screening. In our experience, large kindreds living in relatively closed communities (e.g., the ENG c.817-2A>C kindred with 22 individuals in our study, the majority of them living in the same village) or kindreds with more severely affected individuals (e.g., two cerebral abscesses and a bleeding PAVM in the ENG c.816+5G>A kindred) show more willingness to participate in the family screening.

Demonstrating the ENG/ACVRL1 rate, the Hungarian founder ACVRL1 c.625 + 1G > C variant, 1 of the 34 unique ENG and ACVRL1 variants in our study, accounted for 16.7% (8/48) of the HHT1 + HHT2 kindreds and 23.3% (27/116) of the HHT1 + HHT2 patients at the time of the study.

In our ENG cohort, three variants were found in two families each. The ENG c.817-2A > C families share common ancestors detected by genealogical testing [39], the ENG c.1346del families live 20 km apart, and the ENG c.1687-1G>T kindreds have a shared, otherwise-rare surname. In the ACVRL1 cohort, beyond the above c.625+1G>C founder variant, the c.265T>C and c.1377+2T>A variants were shared by three and two unrelated probands, respectively, originating from neighboring villages in both cases.

The type and intragenic distribution of the ENG and ACVRL1 variants correspond with literature data, with their missense predominance and their predilection to exons 3 and 8 in ACVRL1, and even distribution of variants throughout the extracellular exons, with less missense variants in ENG [4,12,36].

Epistaxis and telangiectasia are, by far, the most frequent clinical manifestations of HHT, with age-related penetrance of both [4,25,41]. Our data confirm this through the epistaxis and telangiectasia prevalence of 93.1% and 76.2% in the overall cohort, respectively. The age-related penetrance is shown by a higher prevalence of the two criteria in the definite compared to the suspected cohorts in both HHT1 and HHT2. The onset of epistaxis precedes telangiectasia [2,41]. Although the onset of symptoms was not assessed in our study, in suspected HHT patients (aged 30.5 years) fulfilling family history plus another criterion, epistaxis was recorded in 90% and telangiectasia in the remaining 10% of cases.

Concerning the overall HHT1 and HHT2 groups, epistaxis was significantly more common in the HHT1 group. The number of asymptomatic mutation carriers in the two groups (0 HHT1 versus 5 HHT2) might account for this difference. In contrast, telangiectasia was significantly less frequent in definite HHT1 compared with HHT2. The age-related onset of symptoms might be the explanation for this [25,41]. In young adults, the coexistence of epistaxis (with earlier onset than in telangiectasia), a congenital AVM and family history is sufficient for categorization as definite HHT. Indeed, when reviewing the fulfilment of clinical criteria, 6 definite HHT1 patients aged 22–37 years were found with epistaxis, PAVM and family history, all lacking telangiectasia for the present.

Our visceral AVM screening protocol corresponds to the international HHT guidelines [42,43]. CT as a PAVM screening method is approved by the HHT guidelines in centers without expertise in transthoracic contrast echocardiography (TTCE) [42]. CT screening of HAVM is also regarded as appropriate in HHT guidelines [42], and furthermore, PAVM and HAVM screenings can be performed simultaneously in this way.

The visceral manifestations observed in our study were compared with others enrolling a minimum of 100 patients. Although the study cohorts (all patients versus adults only), the methodology and the indications (all patients versus symptomatic) of visceral screenings were rather different, PAVMs and CAVMs were significantly more prevalent in HHT1, while HAVMs had a predominance in HHT2 (Table 7). Our results are in accordance with the literature in the case of all three types of visceral AVMs. The prevalence of cerebral abscesses in the PAVM (12.5%) and the overall HHT1 + HHT2 cohorts (3.4%) also corresponds with reported data [44,45]. Gastrointestinal telangiectases show a wide range of overall prevalence in HHT, depending on the indication rather than on the methodology of screening; the majority of these lesions are asymptomatic [46,47]. Symptomatic lesions occur in 11–22% of HHT cases, with non-significant prevalence differences between HHT1 and HHT2 [4,13,37].

A total of 24 pediatric individuals were tested; the family-specific ENG or ACVRL1 variant was detected in 9 of them. Three patients, with a newborn among them (mean age of 2.3 years), were asymptomatic, 5 (aged 10.4 years) had suspected HHT (4 with epistaxis and 1 with telangiectasia plus family history), and the only definite HHT patient was a 12-year-old female. None of them showed symptoms suggestive of visceral lesions. On the other hand, in contrast to the 2011 international HHT guidelines [42] and their 2020 amendments [43], CAVMs were not screened through MR, and the PAVM screening method lacked TTCE. Pediatric CAVM screening is a controversial point in the literature, as it requires sedation or anesthesia [43], the prevalence of CAVMs is relatively low (corresponding with the adulthood CAVM prevalence), and only a subset of them are treated [48]. Thus, in some HHT centers, including ours, screening of asymptomatic individuals for CAVMs is postponed until adulthood [49]. With awareness of the prevalence of congenital HHT visceral lesions, it is conceivable that PAVMs or CAVMs will be detected in a subset of our pediatric patients through the AVM screening at 18 years of age.

The 5 asymptomatic pediatric mutation carriers were phenotypically indistinguishable from the 15 children with wild-type alleles. The confirmation or exclusion of disease in young asymptomatic family members is the main indication for genetic testing in HHT.

Thorough clinical and genetic family screening can reduce the diagnostic time lag in at-risk individuals. In order to do this, a cascade family screening has been developed. Pedigree charts have been constructed for 32 of the 48 kindreds with known variants. The 32 probands have yielded 148 tested and 151 obligately testable at-risk individuals so far. Thus, HHT status is clarified in 180 individuals at present, 24 of whom (13.3%) refused clinical and genetic testing. The cohort of non-testable individuals (*n* = 10) might be somewhat larger, as descendants of wild-type individuals were removed from the pedigree charts in the first years of the study. Furthermore, identification of novel branches on the pedigrees is expected to continually widen the testable cohort.

The main limitation of our study is the incomplete AVM screening in a subset of patients. CAVMs are not screened in childhood, and childhood PAVM screening differs from that of adulthood. TTCE needs to be started in our institution as the first-line PAVM screening method in both children and adults. In addition, a subset of mutation carriers did not give their consent for the visceral AVM screening. Finally, the COVID-19 pandemic, exerting an extreme burden on the Hungarian healthcare system, also hindered the imaging evaluation in a minority of patients.

## 5. Conclusions

We provide the first comprehensive HHT study from Eastern Europe, evaluating 186 individuals from 50 HHT families. Considering the ENG/ACVRL1 variant, family and patient rates and in addition, the distribution of symptoms in HHT1 and HHT2, the genetic and clinical properties of our Hungarian HHT cohort were comparable with literature data. Eleven novel variants were found (5 in the ENG and 6 in the ACVRL1 genes). Both the pitfalls of diagnosing HHT in probands and the significance of a thorough family screening are emphasized in order to reduce the diagnostic time lag in at-risk individuals.

## Figures and Tables

**Table 1:** Novel ENG and ACVRL1 variants detected in this study.

Variant	Protein Change	Type	Population dbSNP 1000 Genomes HMSSP	Co-Segregation	SISA	In Silico Prediction Modeling Software	ACMG §
ENG							
c.360+2insT		SS	none	M: 1D		NA	LP
c.755T>C	p.Ile252Thr	MS	none	M: 2D		PolyPhen2 HumDiv *: possibly damaging PolyPhen2 HumVar *: probably damaging MutPred2 *: non-pathogenic/borderline SIFT **: affecting protein function	VUS
c.836dup	p.Cys279fs	FS	none	M: 1D		NA	LP
c.1133C>T	p.Ala378Val	MS	none	M: 1S I; w: 1NL		PolyPhen2 HumDiv *: possibly damaging PolyPhen2 HumVar *: benign MutPred2 *: non-pathogenic SIFT **: tolerated	VUS
c.1687-1G>T		SS	none	M: 3D + 1S PED	≥87.5%	NA	P
ACVRL1							
c.50del #	p.Leu17Trpfs *2	FS	none			NA	LP
c.89del	p.Pro30Argfs *3	FS	none	M: 1S I		NA	LP
c.997A>T #	p.Ser333Cys	MS	none	M: 4D w: 1NL	≥87.5%	PolyPhen2 HumDiv *: probably damaging PolyPhen2 HumVar *: probably damaging MutPred2 *: likely pathogenic SIFT **: affecting protein function	LP
c.1218G>A #	p.Trp406 *	NS	none	M: 2D w: 2NL		NA	LP
c.1246+1G>C #		SS	none	M: 1D + 1NL I; w: 2NL		NA	LP
c.1462A>C	p.Thr488Pro	MS	none	M: 2D + 1S PED		PolyPhen2 HumDiv *: probably damaging PolyPhen2 HumVar *: probably damaging MutPred2 *: likely pathogenic SIFT **: affecting protein function	VUS

Abbreviations and legend: Under Variant, (#), the specific nucleotides affected in the novel variant are also targets of different, previously described HHT variants [12]. Under variant type (Type), FS: frameshift; MS: missense; NS: nonsense; SS: splice-site. Under Cosegregation, D: clinically definite HHT; (I): incomplete clinical evaluation; M: family-specific mutation; NL: HHT clinically not likely; (PED): pediatric patient; S: clinically suspected HHT; w: wild-type ENG/ACVRL1 allele. Under In Silico Prediction Modeling Software, (*): pathogenic with a score > 0.5; (**): pathogenic with a score < 0.05; NA: not applicable. Under ACMG, (§): pathogenicity was predicted using the Franklin Genoox platform [21]. LP: likely pathogenic; P: pathogenic.

**Table 2:** Demographical data of the HHT1 and HHT2 cohorts.

	HHT1	HHT2	HHT1+HHT2
Overall			
No. (male/female)	53 (31/22)	63 (24/39)	116 (55/61)
Age (years) ± SD	46.1 ± 18.0	43.5 ± 19.9	44.7 ± 19.1
3 or 4 clinical criteria exist			
No. (male/female)	44 (24/20)	47 (14/33)	91 (38/53)
Age (years) ± SD	49.2 ± 16.4	50.0 ± 15.7	49.6 ± 16.1
2 clinical criteria exist			
No. (male/female)	9 (7/2)	11 (7/4)	20 (14/6)
Age (years) ± SD	31.1 ± 17.8	30.0 ± 18.1	30.5 ± 18.0
1 clinical criterion exists			
No. (male/female)	0	5 (3/2)	5 (3/2)
Age (years) ± SD		12.0 ± 11.6	12.0 ± 11.6

Patient age refers to the age at enrollment to our study.

**Table 3:** Clinical manifestations in the HHT1 and HHT2 cohorts based on the Curaçao criteria.

	HHT1	HHT2	*p* Value	HHT1 + HHT2
Epistaxis				
Overall	53/53 (100%)	55/63 (87.3%)	0.007	108/116 (93.1%)
Definite	44/44 (100%)	46/47 (97.9%)	NS	90/91 (98.9%)
Suspected	9/9 (100%)	9/11 (81.8%)	NS	18/20 (90%)
Telangiectasia				
Overall	33/53 (62.3%)	48/63 (76.2%)	NS	81/116 (69.8%)
Definite	33/44 (75%)	46/47 (97.9%)	0.001	79/91 (86.8%)
Suspected	0/9 (0%)	2/11 (18.2%)	NS	2/20 (10%)
PAVM overall ^1^	22/41 (53.7%) ^2^	2/46 (4.3%) ^2^	<0.001	24/87 (27.6%) ^2^
CAVM overall ^3^	4/21 (19.0%) ^2^	1/36 (2.8%) ^2^	NS	5/57 (8.8%) ^2^
HAVM overall ^3^	7/29 (24.1%) ^2^	19/39 (48.7%) ^2^	0.047	26/68 (38.2%) ^2^
GI telangiectasia ^4^	9/53 (17.0%)	7/63 (11.1%) ^2^	NS	16/116 (13.8%) ^2^

Legend: ^1^: PAVM screening was offered to all pediatric and adult patients with mutations and/or fulfilling ≥ 2 diagnostic criteria; ^2^ Only individuals undergoing the screening protocol of the particular visceral organ constitute the denominators; ^3^: CAVM and HAVM screenings were offered to adults only; ^4^ Endoscopic evaluations of upper and/or lower gastrointestinal mucosal telangiectases were performed as needed (see Section 2.2); NS: non-significant.

**Table 4:** Clinical and genetic data of pediatric patients.

Kindreds	Male	Female
HHT1 family members (*n* = 8)		
Individuals with ENG mutation		
3 or 4 clinical criteria exist	-	-
2 clinical criteria exist	12y (E); 13y (E)	-
1 clinical criterion exists	-	-
Wild-type individuals	4y, 11y, 12y	6y, 7y, 17y
HHT2 family members (*n* = 16)		
Individuals with ACVRL1 mutation		
3 or 4 clinical criteria exist	-	9y (E,T)
2 clinical criteria exist	1y (E), 11y (T)	15y (E)
1 clinical criterion exists	0y, 4y	3y
Wild-type individual	5y, 7y, 8y, 11y, 13y	1y, 11y, 11y, 14y

Legend: y: years of age; E: epistaxis; T: telangiectasia.

**Table 5:** Cascade mutation screening: results and future tasks.

Gene	Variant	M+			w	Non-Testable	Refused Screening	Obligately Testable	Facultatively Testable
		≥3 1	2 1	1 1					
ENG	c.111del	1	0	0	2	0	0	0	0
	c.314T>A	1	0	0	1	0	0	2	0
	c.360+1G>A	3	0	0	8	0	1	0	0
	c.816+5G>A §	6	2	0	6	0	7	5	0
	c.817-2A>C (2)	11	1	0	10	0	7	7	0
	c.1134G>A	1	1	0	0	0	0	2	0
	c.1195del	1	0	0	0	0	0	12	2
	c.1346del	1	0	0	1	0	0	2	2
	c.1687-1G>T	1	1	0	0	0	5	0	0
ACVRL1	c.50del	3	1	0	0	0	0	2	2
	c.207C>A	1	0	0	6	7	0	3	0
	c.265T>C (3)	3	0	0	0	0	0	41	3
	c.613del	1	0	0	1	0	0	3	0
	c.625+1G>C (6)	16	4	3	14	2	3	6	0
	c.743_744del	1	0	0	0	0	0	8	0
	c.789C>A	1	1	1	0	0	0	3	0
	c.997A>T	4	0	0	1	1	0	17	7
	c.1120C>T	3	0	0	0	0	0	13	1
	c.1218G>A	2	0	0	2	0	0	2	0
	c.1232G>A	2	0	0	2	0	0	3	3
	c.1246+1G>C	2	0	0	2	0	0	6	4
	c.1280_1291del	1	1	0	0	0	0	8	2
	c.1377+2T>A (2)	3	1	0	4	0	1	6	0
	Total:	69	13	4	60	10	24	151	26
	Screening status	156 clarified					24 refuses	177 testable	

Abbreviations and legend: Under Variant, (§) is a VUS with a ≥ 99.22% probability of cosegregation by SISA; all other variants are pathogenic or likely pathogenic under ACMG Guidelines; the number of kindreds with the same variant is shown in brackets. M: individuals with the family-specific mutation; 1: the number of fulfilled clinical criteria; w: individuals with the wild-type ENG/ACVRL1 alleles. “Non-testable”: the descendants of wild-type individuals in HHT families. “Obligately” or “Facultatively” testable: See Section 2.5 for legends.

**Table 6:** Comparison of the genetic results of some HHT studies evaluating a minimum of 100 patients.

Reference	Population ^1^ HHT Centre ^2^	Molecular Analysis	Probands with Mutations/Total	ENG/ACVRL1 Mutation Rate	ENG/ACVRL1 Family Rate	ENG/ACVRL1 Pt Rate	Pts/Family ^3^ Inds/Family ^4^
[4]	Utah, US ^2^	ENG/ACVRL1 SS	26/34 (76.5%)	14/10 (1.4)	14/12 (1.17)	61/50 (1.22)	111/26 (4.27) ^3^
[29]	Multicentric (US, Australia, Canada, Japan) ^2^	ENG/ACVRL1 SS + 5′UTR SS + LDI	137/200 (68.5%)	71/42 (1.69)	77/50 (1.54)	ND	ND
[30]	Norwegian national study ^1^	ENG/ACVRL1/SMAD4 SS + LDI	total: 105/113 (92.9%) LP+P: 97/113 (85.8%)	total: 30/27 (1.11) LP+P: 30/23 (1.30) SMAD4: 0	total: 42/63 (0.67) LP+P: 39/58 (0.67)	ND	237/97 (2.44) 3 423/113 (3.74) ^4^
[31,32]	French HHT network ^1^	ENG/ACVRL1 SS + 5′UTR SS + LDI, SMAD4 SS	D: 119/136 (87.5%)	D: 42/56 (0.75) SMAD4: 0	D: 40/79 (0.51)		
[25]	French–Italian HHT network ^1^					91/250 (0.36)	343/135 (2.54) ^3^
[33]	Bari, Italy ^2^						135/65 (2.08) ^3^
[34]	Utrecht, The Netherlands ^2^	ENG/ACVRL1 SS + LDI	97/104 (93.3%)	40/31 (1.29)	55/42 (1.31)		
[35]					63/40 (1.58)		508/103 (4.93) ^3^
[36]	Pavia-Crema, Italy ^2^	ENG/ACVRL1 SS	101/137 (73.7%)	26/50 (0.52)	29/72 (0.40)		263/101 (2.60) ^3^ 457/101 (4.52) ^4^
[37]	Spanish RiHHTa registry ^1^	ENG/ACVRL1/SMAD4 SS, NGS + LDI		16/25 (0.64)		36/77 (0.47)	
[13]	Danish national study ^1^	ENG/ACVRL1/SMAD4 SS + LDI	95/107 (88.8%)	29/32 (0.91) SMAD4: 3 fam	47/45 (1.04) SMAD4: 3 fam	151/132 (1.14) SMAD4: 5 pts	320/107 (3.06) ^3^
This study	Debrecen, Hungary ^2^	ENG/ACVRL1/SMAD4 SS, NGS + LDI	48/50 (96%)	18/16 (1.13) SMAD4: 1 fam	21/26 (0.81) SMAD4: 1 fam	53/63 (0.84) SMAD4: 1 pt	121/50 (2.42) ^3^ 186/50 (3.72) ^4^

Abbreviations: ^1^: study population defined; ^2^: HHT center-defined; ^3^: patients/family rate; ^4^: individuals/family rate; D: definite HHT; fam: family; ind: individual; LDI: large deletion/insertion testing; LP: likely pathogenic variant; ND: not detailed; NGS: next-generation sequencing; P: pathogenic variant; pt: patient; SS: Sanger sequencing; 5′UTR: 5′ untranslated region.

**Table 7:** Clinical manifestations of HHT1 and HHT2 in studies evaluating a minimum of 100 patients.

Ref.	Cohort	PAVM		CAVM		HAVM		GI Lesion	
		HHT1	HHT2	HHT1	HHT2	HHT1	HHT2	HHT1	HHT2
[4]	Utah, US ^2^	36/61 (59%)	13/45 (28.9%)	10/61 (16.4%)	1/50 (2%)	1/59 (1.7%)	13/47 (27.7%)	7/39 (18%) bleeding	8/39 (21%) bleeding
		*p* = 0.002		*p* = 0.012		*p* < 0.001			
[29]	Multicentric (US, Australia, Canada, Japan) ^2^	52/77 (67.5%)	24/50 (48%)	7/77 (9.1%)	0	2/77 (2.6%)	7/50 (14%)	7/77 (9.1%)	5/50 (10%)
[25]	French–Italian HHT network ^1^	sy: 32/93 (34.4%)	sy: 13/250 (5.2%)	sy: 2/93 (2.2%)	sy: 3/250 (1.2%)	sy: 0	sy: 19/250 (7.6%)	6/93 (6.5%)	41/250 (16.4%)
		*p* < 0.001		NS		NS		*p* = 0.017	
		asy: 27/50 (54%)	asy: 19/149 (12.8%)	asy: 2/22 (9.1%)	asy: 2/50 (4%)	asy: 20/46 (43.5%)	asy: 87/151 (57.6%)		
		*p* < 0.0001		NS		NS			
[33]	Bari, Italy ^2^	34/45 (75.5%)	34/77 (44.1%)	9/43 (20.9%)	0	27/45 (60%)	64/77 (83.1%)	18/30 (60%)	24/47 (51.1%)
		*p* < 0.0005		*p* < 0.0002		*p* < 0.01		NS	
		Large #: 21/34 (61.8%)	Large #: 6/34 (17.6%)						
		*p* < 0.0001							
[35]	Utrecht, The Netherlands ^2^	167/343 (48.7%)	6/114 (5.3%)	38/260 (14.6%)	1/76 (1.3%)	11/144 (7.6%)	13/32 (40.6%)	56/78 (71.8%)	19/29 (65.5%)
		*p* = 1.2 × 10^−16^		*p* = 0.0015		*p* = 8.7 × 10^−7^		NS	
[37]	Spanish RiHHTa registry ^1^	20/36 (55.5%)	11/77 (14.3%)	3/36 (8.3%)	1/77 (1.3%)	9/36 (25%)	33/77 (42.8%)	Upper: 8/36 (22.2%)	9/77 (11.7%)
		*p* < 0.005		*p* < 0.005		NS, *p* = 0.075		NS	
								Lower: 3/36 (8.3%)	3/77 (3.9%)
[13]	Danish national study ^1^	79/151 (52.3%)	17/132 (12.9%)	2/16 (25%)	1/7 (14.3%)	2/5 (40%)	8/11 (72.7%)	28/151 (18.5%)	15/132 (11.4%)
		*p* < 0.001						NS	
This study	Debrecen, Hungary ^2^	22/41 (53.7%)	2/46 (4.3%)	4/21 (19.0%)	1/36 (2.8%)	7/29 (24.1%)	19/39 (48.7%)	9/53 (17%)	7/63 (11.1%)
		*p* < 0.001		NS, *p* = 0.056		*p* = 0.047		NS	

Abbreviations: ^1^: study population-defined; ^2^: HHT center-defined; (#): A large PAVM is defined as a lesion > 3 mm [33]; asy: asymptomatic; NS: non-significant; sy: symptomatic.

## Data Availability

The data presented in this study are available on request from the corresponding author. The data are not publicly available due to privacy and ethical considerations.
